# Genetic Selection for Growth Rate Reshapes the Plasma Metabolome of Rabbit Does Derived from Vitrified Embryos: Insights into Nutrient Metabolism and Productive Efficiency

**DOI:** 10.3390/vetsci13040391

**Published:** 2026-04-17

**Authors:** Jorge Mateo-López, Alejandro Huertas-Herrera, Mónica Toro-Manríquez, Mette Skou Hedemann, César Cortés-García, Lola Llobat, Diego Páez-Rosas, María Cambra-López, Juan José Pascual, Pablo Jesús Marín-García

**Affiliations:** 1 Department of Animal Production and Health and Food Science and Technology (PASAPTA), Medicine Veterinary Faculty, Universidad Cardenal Herrera-CEU, CEU Universities, 46113 Valencia, Spain; 2 Centro de Investigación en Ecosistemas de la Patagonia (CIEP), Camino Baguales s/n Km 4.7, Coyhaique 5951601, Chile; 3 Department of Animal and Veterinary Sciences, Aarhus University, Blichers Alle 20, DK-8830 Tjele, Denmark; 4 Molecular Mechanisms of Zoonotic Diseases (MMOPS) Research Group, Department of Animal Production and Health and Food Science and Technology (PASAPTA), Medicine Veterinary Faculty, Universidad Cardenal Herrera-CEU, CEU Universities, 46113 Valencia, Spain; 5 Galapagos Science Center, Universidad San Francisco de Quito, Isla San Cristóbal, Islas Galápagos 200150, Ecuador; 6 Fundación Conservando Galápagos, Galapagos Conservancy, Isla Santa Cruz, Islas Galápagos 200350, Ecuador; 7 Dirección del Parque Nacional Galapagos, Unidad Técnica Operativa San Cristóbal, Isla San Cristóbal, Islas Galápagos 200150, Ecuador; 8 Institute for Animal Science and Technology, Universitat Politècnica de València, 46022 Valencia, Spain

**Keywords:** metabolome, efficiency, meat quality, agri-food chain, LPE

## Abstract

In response to the growing global demand for food, intensive genetic selection programs have been implemented to improve livestock efficiency and productivity. Understanding how such selection alters metabolism across nutritional stages is essential for optimizing feeding strategies. In this study, we examined the impact of long-term genetic selection for growth rate (GR) on the plasma metabolome of reproductive female rabbits using an untargeted metabolomics approach. Two vitrified–rederived populations from the same paternal line but separated by 18 generations of GR selection (R19V and R37V) were compared under identical environmental and nutritional conditions. We analyzed 48 plasma samples, showing that GR selection significantly influenced the metabolomic profile. Notably, R37V does exhibited a 76% increase in phospholipid LysoPE (0:0/20:4) concentrations (*p* < 0.0001) than R19V. GR selection affected key metabolites related to lipid metabolism and energy balance, reflecting potential changes in nutrient utilization efficiency. These findings highlight the interplay between genetics and nutrient efficiency in shaping the metabolome, offering insights that may support nutritional management in genetically improved livestock.

## Simple Summary

Long-term genetic selection for growth rate alters the plasma metabolome in reproductive female rabbits. Two populations separated by 18 generations exhibited distinct metabolic profiles under identical conditions. Selection significantly impacted lipid-related pathways and energy metabolism. Notably, a marked increase in specific phospholipids was observed in the highly selected line. These changes suggest improved nutrient utilization efficiency associated with genetic selection. Overall, the findings highlight the interaction between genetics, metabolism, and nutritional physiology.

## 1. Introduction

The meat industry represents a significant sector in global food production, with diverse species contributing to regional consumption. Among these, rabbit meat holds a notable position due to its nutritional value and adaptability to different production systems. In 2021, global rabbit meat production reached 860,000 tons—with a total market value of US$1.5 billion—according to FAOSTAT. Within this context, Spain ranks as the fourth-largest producer of rabbit meat worldwide and holds a prominent position in the sector. Regarding exports, Spain leads with a total of 9300 tons exported in 2021—equivalent to US$32.3 million [1]. Given this scenario, continuing high-quality research aimed at optimizing production efficiency in rabbit meat production appears to be of considerable relevance.

In this framework, nutrition plays a key role in production, as feed conversion ratio (FCR) is the factor with the greatest economic weight [2]. Productivity improvement can be achieved through genetic selection. However, direct selection for FCR is typically performed indirectly via growth rate (GR), due to a negative genetic correlation between FCR and GR. Within the genetic improvement framework, this selection pressure has also driven GR selection resulting in an average increase of +0.84 g/day in daily weight gain per generation (Ayyat et al., 2024; De Rochambeau et al., 1989; Estany et al., 1992; Kasza et al., 2020; Piles & Blasco, 2010) [3,4,5,6,7]. The effects of this selection on meat quality are inconsistent. While some studies report no significant impact on meat quality [8,9], meat quantity [10], or carcass tissues [11], other research suggests that selection for GR may affect meat texture—specifically hardness and chewiness—[12] and decrease the water-holding capacity of raw meat [10].

However, selection for GR may affect more than just meat quality. Potential negative impacts on reproductive traits have been observed in pigs and poultry. In rabbits, selection for GR has not demonstrated adverse effects on reproductive performance or general health status [13]. Hematological and immunological parameters remain largely unaffected in females subjected to selection; however, differential inflammatory responses to *Staphylococcus aureus* infection have been observed across generations [14].

Metabolomics has significantly advanced our understanding of the study of physiological effects in living organisms. This technique has enabled the transition from analyzing a limited number of metabolites to the comprehensive characterization of the entire metabolome. This global approach allows for a deeper and more detailed understanding of the various physiological changes occurring across different species, including rabbits [15,16,17].

Therefore, the objective of this study is to elucidate the metabolomic impact of long-term genetic selection for growth rate (GR) in reproductive female rabbits. Specifically, we compared the metabolic profile of two generations from the same genetic line, separated by 18 generations.

## 2. Materials and Methods

### 2.1. Animal Ethics Statement

The experimental unit consisted of the rabbit doe and its litter. Animals belonged to the paternal R line, which was developed and selected at the UPV. This line originated in 1989 after two generations of random mating involving individuals from three commercial sire lines. Line R is a synthetic population obtained through individual selection based on average daily gain between weaning and slaughter (28 to 63 days of age).

In the present study, two groups from this line were considered, separated by 18 generations of selection: R19V and R37V. A total of 24 animals were used, including 12 individuals from each group [5].

The R19V population was rederived from 256 embryos collected from 25 donor females belonging to eight families of the 18th generation and cryopreserved in 2000. Similarly, the R37V group originated from 301 embryos obtained from 28 donors across nine families of the 36th generation, vitrified in 2015. Consequently, the parental stock of both populations had been preserved as frozen embryos, later thawed and transferred simultaneously in 2015 into recipient females. This approach allowed the generation of contemporaneous populations while minimizing potential cryopreservation effects. After one generation without selection, females from both R19V and R37V lines were obtained at the same time. Further details on embryo cryopreservation and transfer procedures are described elsewhere [18,19].

### 2.2. Experimental Design

Animals were housed at the experimental rabbit farm of the UPV (39.4819° N, 0.3433° W, Valencia, Spain) under standardized environmental conditions, evenly distributed throughout the facility. Ambient temperature ranged from 10 °C to 22 °C throughout the experimental period, with relative humidity maintained between 55 and 70% through an evaporative cooling system (coolers) and mechanical forced ventilation to ensure adequate air exchange. Lighting was artificially controlled at approximately 150 lux at cage level, with a photoperiod of 12 h of light (06:00 to 18:00 h) and 12 h of darkness. The experiment was conducted between March 2019 and January 2020. The incorporation of animals from each generation was balanced over time.

At 63 days of age, females from both groups were individually housed in breeding cages (700 × 500 × 320 mm). Artificial insemination was performed at 19 weeks of age using semen from males of the corresponding generation. From day 28 of gestation until weaning, females were provided with an external nest box (220 × 350 × 370 mm). The reproductive variables recorded included total number of kits born, number of kits born alive, litter size at weaning, and litter weight at birth and at weaning. Although the reproductive variables recorded included total number of kits born, number of kits born alive, litter size at weaning, and litter weight at birth and at weaning, these data were recorded not for analysis, but to standardize experimental conditions

Up to the first parturition, all animals were fed the same commercial diet formulated for young reproductive rabbits, offered ad libitum (9.9 MJ digestible energy, 120 g digestible protein, and 480 g neutral detergent fiber per kg dry matter). No antibiotics or other medications were administered in the feed or drinking water during the experimental period. Blood samples were collected at first parturition. A total of 48 samples were obtained from the central ear artery (1 mL in EDTA tubes), immediately centrifuged at 700× *g* for 5 min, and plasma was stored at −80 °C until analysis. Metabolomic analyses were performed in 2024, and statistical data analyses were conducted in 2025. To evaluate whether genetic selection for growth rate affected the plasma metabolome, plasma samples from females belonging to the different genetic groups were analyzed using a metabolomic approach, and the resulting metabolic profiles were compared between groups. From parturition to weaning (28 days postpartum), females received a commercial diet for adult reproductive rabbits ad libitum (12.3 MJ digestible energy, 148 g digestible protein, and 359 g neutral detergent fiber per kg dry matter).

Animal welfare and health status were monitored daily by trained personnel through visual inspection of behavior, feed and water intake, body condition, and the presence of clinical signs of disease or injury. Animals showing signs of illness or abnormal behavior were evaluated by veterinary staff according to the farm health management protocol.

### 2.3. Plasma Metabolomic Analysis by LC-MS

#### 2.3.1. Solvents and Chemical Standards for Metabolomic Analysis

All plasma samples were subjected to untargeted metabolomic profiling. Solvents included HPLC-grade acetonitrile (VWR, West Chester, PA, USA), formic acid (FA, Merck KGaA, Darmstadt, Germany), and Milli-Q water. Internal standards added during sample preparation were glycocholic acid (glycine-1-13C) and 4-chloro-DL-phenylalanine (Merck KGaA, Darmstadt, Germany).

#### 2.3.2. Sample Preparation and LC-MS Analysis

Each plasma sample was processed individually at the Aarhus University laboratory (Foulum, ISO 17045 accredited). Protein precipitation was performed by adding 450 µL of ice-cold acetonitrile to 150 µL of plasma, including internal standards at a final concentration of 0.01 mg/mL. Samples were prepared in 1 mL 96-well plates, mixed for 1 min, incubated at 4 °C for 10 min, and centrifuged at 2250× *g* for 25 min at 4 °C.

Approximately 400 µL of supernatant was transferred to 96-well filter plates (Phenomenex), followed by vacuum filtration. The filtrate was then distributed into two 96-well plates (65 µL per well) and evaporated to dryness under vacuum (≈2.5 h at 30 °C, 805× *g*). Residues were reconstituted in H_2_O:ACN:FA (95:5:0.1, *v*/*v*/*v*) using the original volume. Plates were sealed and centrifuged again under the same conditions.

Chromatographic analysis was carried out using an UHPLC system (Nexera X2 LC) coupled to an LCMS-9030 Q-TOF mass spectrometer (Shimadzu, Kyoto, Japan), operating in both positive and negative electrospray ionization modes. Separation was achieved using an Acquity HSS T3 column (1.7 µm, 100 × 2.1 mm). The column temperature was maintained at 40 °C, while samples were kept at 10 °C. Injection volume was 3 µL.

A binary gradient system was employed with solvent A (water + 0.1% formic acid) and solvent B (acetonitrile + 0.1% formic acid) at a flow rate of 0.4 mL/min. The gradient increased linearly from 5% to 100% B over 12 min, followed by a 1 min hold and a 3 min re-equilibration, resulting in a total run time of 16 min.

Mass spectra were acquired in full scan (MS) and auto MS/MS modes across a range of 50–1000 *m*/*z*. Instrument settings included: ion source temperature 300 °C, capillary temperature 250 °C, heat block 400 °C, spray voltage −3.5 kV (ESI−), nebulizer gas 3 L/min, drying gas 10 L/min, and detector voltage 2.02 kV. MS/MS acquisition was performed using data-dependent analysis with up to 10 precursor ions per cycle and collision energy of 20 eV (±10 eV). External calibration was performed using sodium iodide solution (400 ppm in methanol). Data acquisition was carried out using LabSolutions software (version 5.114).

#### 2.3.3. Sample Quality Control and Metabolomics Data Pre-Processing

Quality control (QC) samples were prepared by pooling aliquots of all samples and processed identically. QCs were injected periodically throughout the analytical sequence and used to monitor system stability and correct signal drift. Blank samples were also analyzed to detect potential contaminants and carryover. Sample order was randomized to avoid systematic bias.

Data processing, including peak detection, alignment, and gap filling, was performed using MS-DIAL (accessed on 15 December 2024) software, with metabolite annotation based on its integrated library. The resulting data matrix was exported to Excel and filtered to remove peaks detected in blanks. Only signals within the chromatographic retention window and below 700 *m*/*z* were retained.

Data were mean-normalized and autoscaled before PCA and PLS-DA. Principal component analysis (PCA) was initially conducted using MetaboAnalyst (accessed on 1 February 2025) to assess data quality and identify outliers. Subsequently, partial least squares discriminant analysis (PLS-DA) was applied to identify metabolites differentiating the groups. Model validation was performed using repeated random subsampling. Model performance was evaluated using R^2^ and Q^2^ values, and relevant variables were selected based on variable importance in projection (VIP) scores. Cross-validation was carried out using a five-fold method with a maximum of five components. The potential for overfitting in PLS-DA models was carefully considered. In our study, the models exhibited average R^2^ and Q^2^ values of 0.87 and 0.57, respectively. The difference between R^2^ and Q^2^ (~0.3) falls within commonly accepted thresholds for untargeted metabolomics studies.

#### 2.3.4. Metabolite Identification

Metabolite identification was achieved by querying the Human Metabolome Database (HMDB, accessed on 15 February 2025) based on accurate mass measurements, fragmentation patterns, and LC-MS data. Metabolite annotation was performed following the guidelines of the Chemical Analysis Working Group of the Metabolomics Standards Initiative [20]. Metabolites identified at level 1 were confirmed by matching accurate mass (*m*/*z*), retention time, and MS/MS spectra with commercially available standards. When standards were not available, metabolites were putatively annotated (level 2) if their *m*/*z* and MS/MS spectra matched entries in the HMDB or METLIN (accessed on 15 March 2025), databases or putatively characterized (level 3) if only *m*/*z* values were matched. Molecular features without annotation (level 4) correspond to less confident classifications. In this paper, only level 2 was used.

#### 2.3.5. Statistical Analysis of Metabolites

Metabolites showing differences between genetic lines were analyzed after confirming normal distribution using a generalized linear model (GLM) implemented in SAS 9.4. Least-square means were compared using *t*-tests. All experimental units within each genetic group were maintained under homogeneous and standardized conditions, minimizing variability and reducing the likelihood of potential confounding factors. To account for multiple comparisons inherent in high-dimensional metabolomics data, *p*-values were adjusted using the Benjamini–Hochberg false discovery rate (FDR) procedure. Statistical differences were declared at a significance level of FDR-adjusted *p* < 0.05, ensuring that the reported differential metabolites reflect robust and reliable biological differences.

Hierarchical clustering heatmaps (MetaboAnalyst, accessed on 1 April 2026) were generated to visualize the relative abundance of metabolites across plasma samples. Normalized metabolomics data were autoscaled (mean-centered and divided by the standard deviation for each feature). Pairwise distances between features were calculated using Pearson correlation, and hierarchical clustering was performed using the Ward method. The top 2000 features, selected based on interquartile range (IQR), were initially considered, and subsequently, the 25 most significant metabolites were selected based on ANOVA for detailed visualization. Heatmaps display metabolite abundances with a color gradient, where red indicates higher abundance and blue indicates lower abundance. Sample and feature labels were shown, with font size set to 8 for readability.

## 3. Results

**Figure 1:**
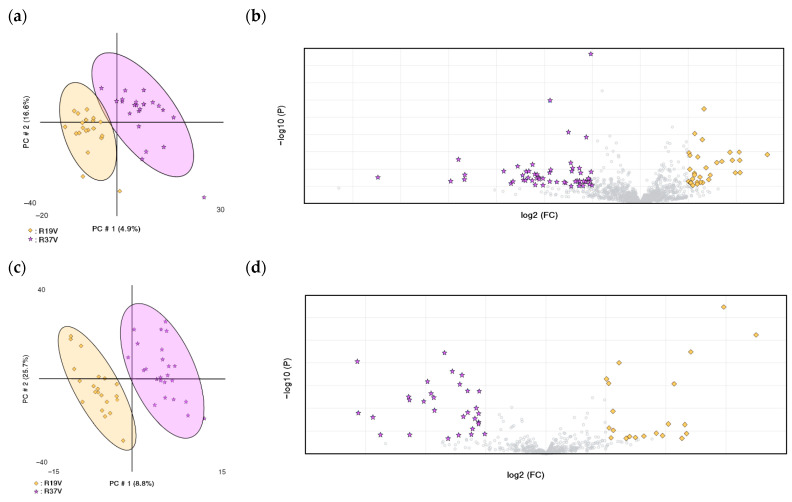
Summary of the results of the untargeted metabolomics assay regarding the effect of genetic selection for growth rate in rabbits does comparing 18 generations of selection (R19V vs. R37V) (**a**,**c**). Partial Least Squares Discriminant Analysis (PLS-DA) score plot of plasma in positive (**a**) and negative mode (**c**). The colors and shapes correspond to the two experimental groups: 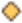: R19V, 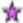: R37V. (R^2^ = 0.85, Q^2^ = 0.37; R^2^ = 0.90, Q^2^ = 0.76, for (**a**) and (**c**), respectively). (**b**,**d**). Volcano plots show significantly different abundant metabolites between both experimental groups (two-sided Wilcoxon rank tests with the value adjusted by false discovery rate, FDR < 0.05) are shown; fold change threshold >2.0 in the volcano plots. Volcano plots are in positive (**b**) and in negative mode (**d**).

Figure 1 illustrates the effect of genetic selection for GR, comparing 18 generations of selection on the PLS-DA score plots derived from the non-targeted metabolomics analysis. Figure 1a and Figure 1c show the first two principal components of the PLS-DA model in positive (2a) and negative (2c) ionization modes, explaining 21.5% and 34.5% of the total variance, respectively. These components effectively differentiate the experimental groups, with minimal overlap observed in the positive mode and no overlap in the negative mode. The models demonstrated average R^2^ and Q^2^ values of 0.87290 and 0.56690, respectively. Figure 1b,d present volcano plots highlighting the key metabolites contributing to the PLS-DA-based separation. After the identification, Table 1 summarizes the tentative metabolites that explain the differences between generations. R37V displayed higher plasmatic levels of LysoPE (0:0/20:4) (LPE) (modified phospholipid) (+76%; *p* < 0.0001) than R19V animals.

**Figure 2:**
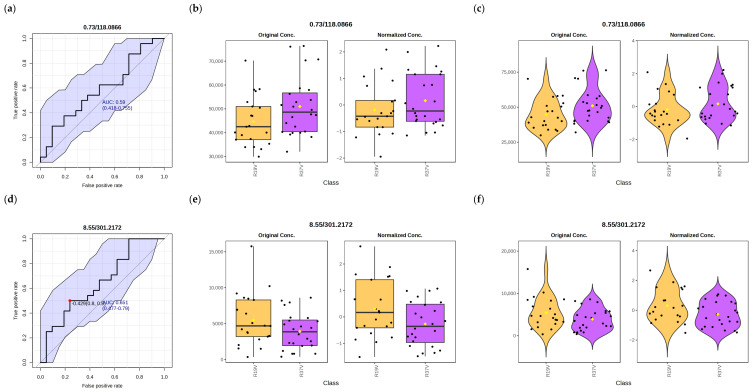

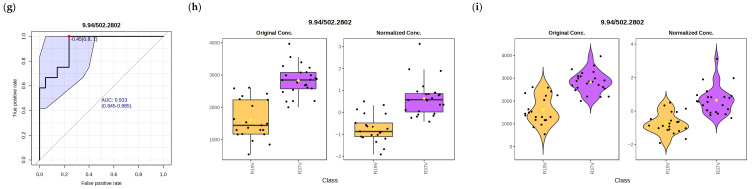
Evaluation of discriminative performance and abundance patterns of key metabolites between genetic line. (**a**–**c**) Betaine, (**d**–**f**) Kaurane diterpenoid, and (**g**–**i**) LysoPE(0:0/20:4). For each metabolite, ROC curves are shown in the first panel of the set (**a**,**d**,**g**), box plots in the second (**b**,**e**,**h**), and violin plots in the third (**c**,**f**,**i**), illustrating both the discriminative ability and the abundance patterns across experimental groups. The colors and shapes correspond to the two experimental groups: 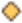: R19V.

Figure 2 illustrates the evaluation of the discriminant performance and abundance patterns of the key metabolites. Receiver Operating Characteristic (ROC) curves (a, d, g) and distribution plots—box (b, e, h) and violin (c, f, i)—are shown for the metabolites that most effectively discriminate between experimental groups. The Area Under the Curve (AUC) values were different, high, with an average exceeding 0.75, indicating a strong discriminant capacity of these metabolites in distinguishing between normal and thermally stressed animals. The discriminant capacity was particularly high for LysoPE(0:0/20:4), with a mean value of 0.933, ranging from 0.845 to 0.985, indicating a strong ability of this metabolite to distinguish between normal and thermally stressed animals.

**Figure 3:**
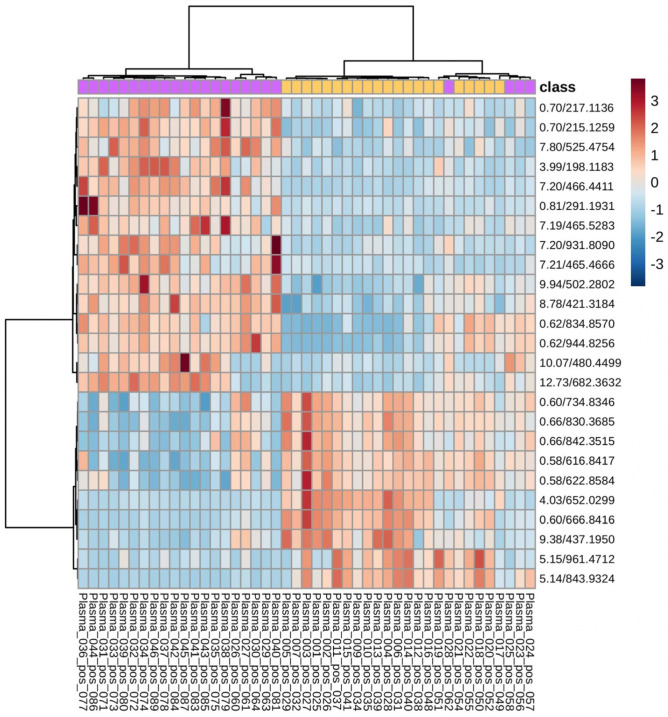
Heatmap showing relative abundance of the top 2000 autoscaled metabolites across plasma samples. Samples and metabolites were clustered using Pearson correlation and Ward’s method. Color scale indicates relative abundance, with red representing higher abundance and blue lower abundance. Class annotation at the top indicates experimental groups. The colors correspond to the two experimental groups: yellow: R19V, purple: R37V.

Figure 3 shows the hierarchical clustering heatmap; it reveals distinct patterns of metabolite abundance among the plasma samples. Two major clusters of samples were observed, corresponding to the different experimental groups, as indicated by the class annotation bar. Metabolites also formed clusters based on similar abundance patterns, suggesting groups of compounds that vary together across samples. Notably, several metabolites showed high abundance in one group (red) and low abundance in the other (blue).

## 4. Discussion

Although genetic selection for GR has been widely implemented in livestock species, its long-term metabolic consequences—particularly during critical physiological stages—remain largely unexplored. In this study, we used untargeted metabolomics approach to assess how 18 generations of selection for GR have influenced the metabolomic profile of reproductive rabbit does. Our findings revealed specific metabolic shifts associated with selection for GR, providing insights into the physiological adaptations and potential trade-offs in reproductive does.

Long-term genetic selection for GR had a pronounced impact on the metabolome, as evidenced by the clear separation between experimental groups—both globally and within each physiological stage—and by significant differences in the concentration of specific metabolites. Due to the novelty of this approach, no studies were found in the reviewed literature that directly examine metabolomic changes resulting from genetic selection through contemporaneous comparisons of individuals from different generations. Nevertheless, a study in *Crassostrea gigas* reported distinct metabolic profiles between selectively bred and wild individuals, identifying metabolic signatures associated with artificial selection for fast growth. Key altered pathways include CoA biosynthesis, steroid hormone biosynthesis, and amino acid metabolism, suggesting a metabolic basis for the enhanced growth in selected oysters [21]. In a different selective context, research in *Drosophila melanogaster* showed that selection for stress tolerance induces widespread metabolomic changes, accompanied by correlated expression of general stress response genes. These findings suggest a degree of coordinated regulation between metabolite levels and gene expression under selective pressure [22]. Additionally, other studies have identified genetic factors influencing the metabolome across different pig breeds, providing evidence of an underlying genetic basis for the metabolic profile [23]. Some studies have identified distinct metabolic profiles in rabbit does, although these were not obtained through metabolomic analyses [24]. Nevertheless, other studies have reported changes in the levels of specific metabolites in relation to Grin different species [23,25,26].

Next, we will discuss the only metabolite that showed significant differences between the experimental groups comparing 18 generations of selection for GR. LPE was the only identified plasma metabolite that consistently showed significant concentration changes. This metabolite emerged as the most consistent biomarker associated with GR selection.

LPE is a phospholipid metabolite from the cephalin group involved in membrane fluidity. It is generated by phospholipase A activity on phosphatidylethanolamine [27].

LPE can be further metabolized in various tissues into lecithin and phosphatidylethanolamine, both of which serve as precursors of cellular phosphoglycerides [28]. LPE has been described with association with GR [29]. Our data are concordant with other studies where an increased concentration of most phospholipids and cholesterol was observed in the most selected animals [30]. Thus, the literature indicates that LPE plays a key role in glycerophospholipid metabolism, acting as an intermediate in the degradation and remodeling of various phospholipids. Additionally, it functions as a signaling molecule involved in processes such as phagocytosis, apoptosis, cellular stress response, membrane homeostasis, and vesicle formation. The elevated LPE levels in R37V—the more selected line, at both time points, despite sharing the same diet as the less selected group—suggest a genetically driven nutritional metabolic adaptation. This alteration in lipid metabolism could reflect an increased capacity to mobilize and remodel membrane lipids, optimizing the use of dietary fats to meet higher energetic and functional demands [28,31,32,33,34,35,36,37].

This study underscores how genetic selection and physiological stage jointly influence the metabolome of reproductive female rabbits. The consistent change in LPE points to lipid metabolism as a key target of selection. These insights pave the way for deeper molecular studies on the metabolic basis of genetic improvement.

Our findings revealed specific metabolic shifts associated with long-term selection for growth rate, providing insights into the physiological adaptations in reproductive rabbit does. Similar metabolomic studies in livestock species, such as pigs and chickens, have reported changes linked to genetic breeding, supporting the relevance of our observations [38,39]. Nevertheless, this study is limited by its focus on plasma metabolomics, sample size, and a single rabbit population, which may not capture tissue-specific effects or generalize across breeds. Despite these limitations, our results contribute to understanding the metabolic consequences of genetic selection and help test the hypothesis that prolonged selection for growth rate impacts reproductive metabolism.

## 5. Conclusions

This study aimed to investigate the effects of long-term genetic selection for growth rate (GR) on the metabolomic profile of reproductive female rabbits. The main conclusions are as follows: (i) Genetic selection for GR is associated with changes in certain metabolites. (ii) Among the affected metabolites, lysophosphatidylethanolamine (LPE) consistently emerged as the most altered across comparisons. (iii) These observations suggest potential metabolic shifts related to lipid metabolism that could reflect physiological adaptations linked to selection for GR. Further targeted studies, including transcriptomic or proteomic integration, are needed to clarify the underlying regulatory pathways and to assess possible trade-offs.

## Figures and Tables

**Table 1:** List of plasma metabolites differentiating among genetic selection for growth rate in rabbits does.

			R19V *		R37V *			
RT-*m*/*z*	ION	Metabolite	X	±SE **	X	±SE	Fold ****	*p*_Value
0.73/118.0866	[M+H]^+^	Betaine	44,649	±2506	50,969	±2344	0.8760	0.0724
8.55/301.2172	[M+H]^+^	Kaurane diterpenoid ***	5461	±679	3921	±635	1.3928	0.0511
9.94/502.2802	[M+H]^+^	LysoPE(0:0/20:4)	1608	±116	2834	±109	0.5674	<0.0001

(*) R19V and R37V: contemporary populations of the R line, rederived from vitrified embryos of generations 19 and 37, respectively. (**) LS means ± Standard error (***) Ent-6,16-Kauradien-19-oic acid. (****) Fold change was calculated by dividing the mean intensity of the plasma metabolite in R19V by the mean intensity of the corresponding metabolite in R37V animals.

## Data Availability

The raw data supporting the conclusions of this article will be made available by the authors on request.

## Abbreviations

The following abbreviations are used in this manuscript:

ACN	Acetonitrile
AUC	Area Under the Curve
ESI	Electrospray Ionization
FA	Formic Acid
FCR	Feed Conversion Ratio
GLM	Generalized Linear Model
GR	Growth Rate
HMDB	Human Metabolome Database
LC-MS	Liquid Chromatography–Mass Spectrometry
LPE	Lysophosphatidylethanolamine
LysoPE	Lysophosphatidylethanolamine
MJ	Megajoule
MS	Mass Spectrometry
MS/MS	Tandem Mass Spectrometry
*m*/*z*	Mass-to-charge ratio
PCA	Principal Component Analysis
PLS-DA	Partial Least Squares Discriminant Analysis
ppm	Parts Per Million
QC	Quality Control
Q-TOF	Quadrupole Time-of-Flight
ROC	Receiver Operating Characteristic
RT	Retention Time
SE	Standard Error
UHPLC	Ultra-High-Performance Liquid Chromatography
UPV	Universitat Politècnica de València
VIP	Variable Importance in Projection
